# Evaluation of BD Phoenix CPO Detect and NG-Test CARBA 5 for the detection and classification of carbapenemase-producing *Enterobacterales* using whole-genome sequencing as the reference standard

**DOI:** 10.1128/spectrum.00890-26

**Published:** 2026-06-18

**Authors:** Nisha Aji, Namitha Thomas, Andrés Pérez-López, Mohammed Suleiman

**Affiliations:** 1 Department of Pathology, Sidra Medicine, Doha, Qatar; 2 Department of Pathology and Laboratory Medicine, Weill Cornell Medicine in Qatar, Doha, Qatar; 3 Department of Biomedical Sciences, Qatar University 61780, Doha, Qatar; The George Washington University School of Medicine and Health Sciences, Washington, DC, USA

**Keywords:** carbapenemases, CPE, BD Phoenix CPO Detect, NG Carba 5

## Abstract

Rapid detection and accurate classification of carbapenemase-producing *Enterobacterales* (CPE) are essential for antimicrobial stewardship and infection prevention. We evaluated the performance of the BD Phoenix CPO Detect (BD-CPO) assay and the NG-Test CARBA 5 (NG-C5) using whole-genome sequencing (WGS) as the reference standard in a tertiary pediatric center. A total of 161 non-duplicate WGS-confirmed CPE isolates recovered from clinical and screening specimens were included. NG-C5 demonstrated a sensitivity of 99.4%, correctly identifying and classifying carbapenemase genes in 160 of 161 isolates, including those harboring dual carbapenemase genes. BD-CPO showed a sensitivity of 96.3% (155/161) for CPE detection; however, accurate Ambler class assignment was achieved in only 70.2% (113/161) of isolates. Misclassification occurred in 9.3% (15/161) of isolates, and six false-negative results (3.7%) were observed with the BD-CPO test, particularly among isolates harboring *bla*_OXA_ genes. Both assays provide rapid detection of carbapenemase production compared to conventional phenotypic methods. However, NG-C5 demonstrated superior performance in carbapenemase gene identification and classification. Implementation of rapid carbapenemase detection assays can support timely therapeutic optimization and infection control interventions, although the results should be interpreted within a comprehensive diagnostic framework that includes molecular confirmation and full antimicrobial susceptibility testing.

**IMPORTANCE:**

Accurate detection of carbapenemase-producing Enterobacterales (CPE) is critical for guiding targeted antimicrobial therapy and implementing effective infection control strategies. Clinical microbiology laboratories rely on a variety of phenotypic and rapid diagnostic methods; however, their ability to correctly detect and classify carbapenemases remains variable. In this study, we compared the performance of the BD Phoenix CPO Detect and NG-Test CARBA 5 using whole-genome sequencing as the reference standard. Our results demonstrate important differences in diagnostic accuracy, particularly for OXA-type carbapenemases, providing practical evidence to guide laboratories in selecting reliable tools for rapid CPE detection and classification.

## INTRODUCTION

Carbapenemase-producing *Enterobacterales* (CPE) represent a major global public health threat, associated with high mortality, limited treatment options, and substantial healthcare costs (1). CPE harbor genes encoding carbapenemase enzymes that hydrolyze most β-lactam antibiotics, including carbapenems, which are often considered last-resort agents for the treatment of infections caused by multidrug-resistant bacteria (2, 3). CPE infections pose a particular challenge in healthcare settings due to their capacity for rapid transmission and limited therapeutic options (4). Transmission reservoirs include colonized or infected patients, environmental surfaces, medical devices, and the hands of healthcare workers (5, 6). Risk factors for acquisition include prolonged intensive care unit stays, immunosuppression, and prior broad-spectrum antibiotic exposure (5, 7). Accurate laboratory detection is therefore essential for both appropriate antimicrobial therapy and effective infection prevention and control.

Carbapenemases are categorized into three main classes: serine β-lactamases (Ambler class A, including KPC enzymes), metallo-β-lactamases (MBLs) (class B, including NDM, VIM, and IMP variants), and OXA-type enzymes (class D) (8). Detecting the production of these enzymes using conventional antimicrobial susceptibility testing (AST) alone is challenging, as minimum inhibitory concentrations (MICs) for carbapenems can vary widely depending on enzyme expression, hydrolytic efficiency, and coexisting carbapenem resistance mechanisms such as porin loss or efflux pump overexpression (8, 9). Consequently, laboratories employ a range of phenotypic, immunochromatographic, and molecular methods for CPE detection. Molecular methods, including syndromic panels, are increasingly used in clinical laboratories and detect resistance genes directly from specimens; however, they do not assess gene expression or enzyme activity, which may limit their ability to fully predict phenotypic resistance (8, 9).

The BD Phoenix CPO Detect test (BD-CPO) (Becton, Dickinson, and Company, Franklin Lakes, NJ, USA) is integrated within the Gram-negative MIC panel and provides antimicrobial susceptibility results alongside functional detection of carbapenemase activity based on enzymatic hydrolysis, thereby representing a phenotypic assay (10). Rapid immunochromatographic assays such as NG-Test CARBA 5 (NG-C5) (NG Biotech, Guipry, France) are antibody-based lateral flow immunoassays for the detection of carbapenemase enzymes, enabling rapid and specific identification of the most prevalent carbapenemases (KPC, NDM, VIM, IMP, and OXA-48-like) within minutes (11). Whole-genome sequencing (WGS) serves as the genomic reference standard, providing comprehensive antimicrobial resistance gene profiling and high-resolution strain typing; however, it reflects gene carriage and does not provide information on gene expression or enzyme activity (12).

However, comparative real-world evaluations of these methods, particularly in pediatric populations, remain limited. This study aimed to compare the performance of the BD-CPO and NG-C5 against WGS for the detection and classification of CPE in a tertiary-care pediatric setting.

## MATERIALS AND METHODS

### Study design and setting

A retrospective study was conducted at Sidra Medicine, a tertiary-care pediatric hospital in Qatar, from February 2021 through September 2025. The patient population was diverse with respect to nationality, clinical presentation, medical history, and underlying conditions. Non-duplicate CPE isolates recovered from both clinical and screening specimens were included. All isolates were identified to the species level using matrix-assisted laser desorption ionization-time of flight mass spectrometry (MALDI-TOF MS) (Bruker Daltonik GmbH, Bremen, Germany). Antimicrobial susceptibility testing was performed using the BD Phoenix M50 system with the gram-negative NMIC-501 panel (Becton, Dickinson, and Company, Franklin Lakes, NJ, USA), which incorporates the BD-CPO test. Isolates were also tested using the NG-C5 assay and WGS.

### Diagnostic tests

#### BD-CPO

The BD-CPO (Becton, Dickinson, and Company, Franklin Lakes, NJ, USA) provides phenotypic detection of carbapenemase production and assigns Ambler classification (class A, B, or D). Testing was performed according to the manufacturer’s instructions. Briefly, colonies were suspended in BD Phoenix ID broth and adjusted to a turbidity of approximately 0.25 McFarland. Automated inoculum preparation and standardization were performed using the BD Phoenix AP instrument in conjunction with the BD Phoenix M50 system. The standardized suspension was inoculated into the appropriate AST panel and loaded into the Phoenix instrument. The system automatically determines MICs, detects carbapenemase production, and assigns the Ambler class based on panel-specific algorithms. Antimicrobial susceptibility results were interpreted according to the Clinical and Laboratory Standards Institute (CLSI) M100 performance standards (13).

#### NG-C5

The NG-C5 (NG Biotech, Guipry, France) is a multiplex immunochromatographic lateral flow assay for the qualitative detection and differentiation of the five most prevalent carbapenemase families (KPC, OXA-48-like, VIM, IMP, and NDM) from pure colonies of *Enterobacterales*. Testing was performed using pure colonies from overnight cultures grown on blood agar plates and MacConkey agar (Oxoid, Basingstoke, UK) or CHROMagar SuperCARBA (CHROMagar, Paris, France). According to the manufacturer’s instructions, 3–5 colonies were suspended in 150 µL of extraction buffer and vortexed thoroughly. Subsequently, 100 µL of the suspension was applied to the sample well of the test cassette (14). The results were interpreted after 15 min. The test was considered valid only if the control line appeared. The presence of any test line in addition to the control line indicated a positive result for the corresponding carbapenemase family (14).

### Molecular confirmation by WGS

Genomic DNA was extracted from bacterial colonies using the EZ1 Advanced XL platform (QIAGEN, Hilden, Germany). DNA libraries were prepared using the Nextera XT library preparation kit (Illumina, San Diego, CA, USA) and sequenced on the Illumina MiSeq platform using 2 × 300 bp paired-end chemistry. Antimicrobial resistance (AMR) genes were identified using AMRFinderPlus v3.11.20 (15), and plasmid replicons were predicted using MobSuite v3.1.9 (16), both implemented within the SOLU bioinformatics platform (Solugenomics, https://www.solugenomics.com) using default parameters.

### Statistical analysis

BD-CPO and NG-C5 results were collected and compared with those obtained by the WGS reference method. Data were recorded and managed using Microsoft Excel 2024. Only sensitivity was calculated to assess diagnostic performance, as the study included only CPE-positive isolates. Additional clinical variables, including patient location, culture source (clinical versus screening), and antimicrobial susceptibility results, were collected.

## RESULTS

### General characteristics of the tested isolates

A total of 161 non-duplicate CPE isolates characterized by WGS from pediatric inpatient units were included in this study. Of these, 84.5% (136/161) were recovered from screening samples, whereas 15.5% (25/161) were recovered from clinical samples. *Escherichia coli* (110/161, 68.3%) was the most commonly isolated species, followed by *Klebsiella* spp. (*Klebsiella pneumoniae* [43/161], 26.7% and 1 *Klebsiella oxytoca* [1/161, 0.6%], *Citrobacter* spp. [4/161, 2.5%], and *Enterobacter* spp. [3/161, 1.9%]). A single carbapenemase-encoding gene was detected in 147 (91.3%) isolates, whereas 14 (8.7%) carried two genes. Among the 161 CPE, 122 (75.8%) carried *bla*_NDM_, 50 (31.1%) *bla*_OXA_, and 3 (1.9%) *bla*_VIM_; these counts include isolates harboring dual carbapenemase genes.

**Fig 1:**
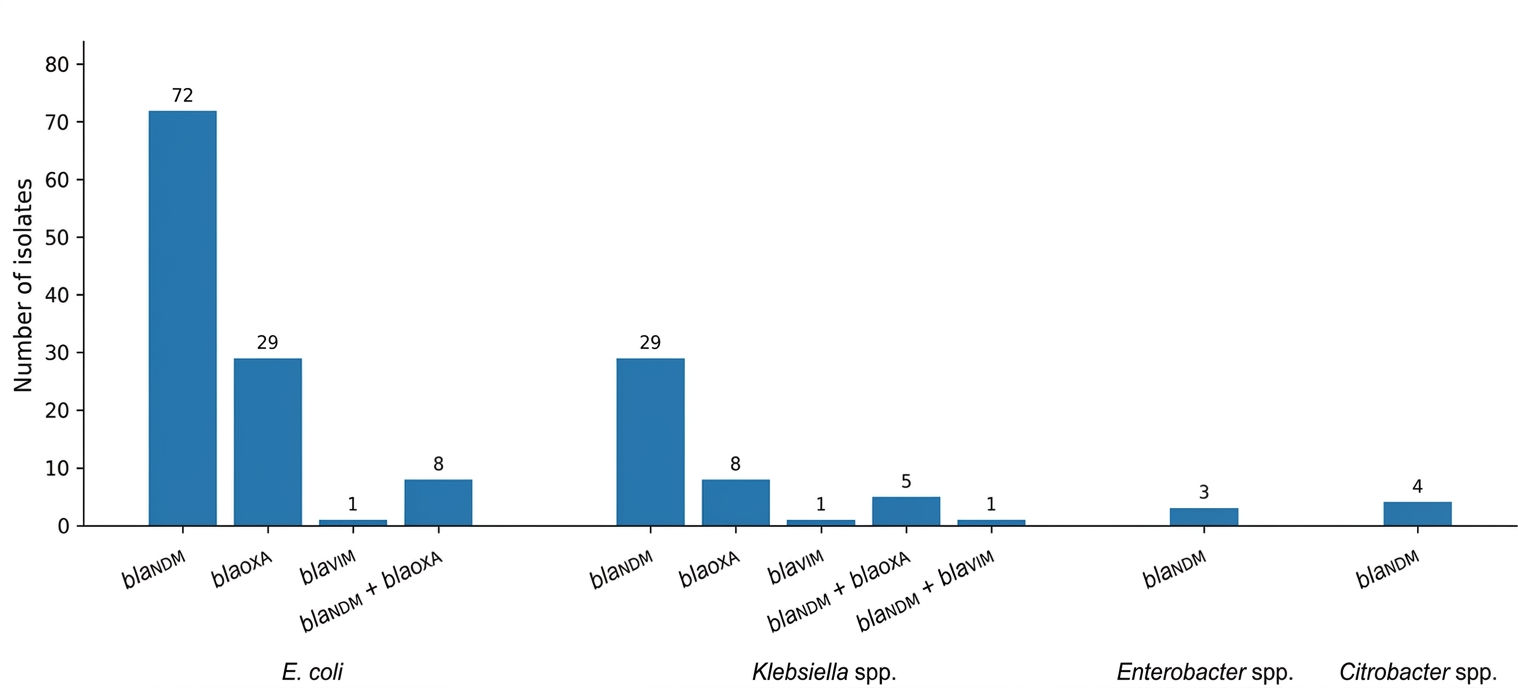
Distribution of carbapenemase reference genes among the CPE isolates as characterized by WGS. NDM, New Delhi Metallo-β-lactamase; OXA, OXA-type β-lactamases; VIM, Verona integron-encoded metallo-β-lactamase.

The distribution of carbapenemase genes among the CPE isolates is shown in Fig. 1.

### Performance characteristics of NG-C5

The NG-C5 assay demonstrated a sensitivity of 99.4%, correctly identifying and classifying isolates into the appropriate Ambler class in 160 of 161 cases. Notably, NG-C5 was able to detect two carbapenemase enzymes simultaneously in 14 CPE isolates. One discrepancy was observed: an *E. coli* isolate harboring *bla*_NDM-5_ by WGS was misidentified as NDM + KPC by NG-C5. The performance of NG-C5 compared with WGS is shown in Table 1. Detailed isolate-level detection and classification results are provided in Table S1 in the supplemental material.

**TABLE 1:** Performance of NG-C5 compared with WGS^*b*^

WGS carbapenemase category	No. of isolates	Correctly identified, *n* (%)	Misidentified, *n* (%)
NDM only (all variants)	108	107 (99.1)	1 (0.9)^*a*^
OXA-48–like only	37	37 (100)	0
VIM only	2	2 (100)	0
Dual carbapenemases (NDM + OXA or NDM + VIM)	14	14 (100)	0
Total	161	160 (99.4)	1 (0.6)

^*a*^ One *E. coli* isolate harboring *bla*_NDM-5_ by WGS was misidentified as NDM + KPC by NG-C5.

^*b*^ NDM, New Delhi metallo-β-lactamase; NG-C5, NG-Test CARBA 5; OXA-48–like, OXA-48 family carbapenemases; VIM, Verona integron-encoded metallo-β-lactamase; WGS, whole-genome sequencing.

### Performance characteristics of BD-CPO and susceptibility results

The BD-CPO assay demonstrated a sensitivity of 96.3% (155/161) for the detection of CPE. However, only 70.2% (113/161) were accurately classified into Ambler classes. class B carbapenemases were correctly identified in 83.6% (92/110) of isolates, predominantly involving *bla*_NDM_ variants. Class D carbapenemases were correctly identified in 64.0% (32/50) of isolates. In 16.8% (27/161) of isolates, the panel detected carbapenemase production but failed to assign a specific Ambler class. In 3.7% (6/161) of isolates (all *E. coli* harboring *bla*_OXA_), the BD-CPO failed to detect carbapenemase production, representing false-negative results. In addition, incorrect Ambler class assignment (misclassification) was observed in 9.3% (15/161) of isolates.

Among the 161 CPE isolates, 124 isolates harboring *bla*_NDM_, *bla*_VIM_, or dual carbapenemases were intermediate or resistant to all tested carbapenems and to ceftazidime-avibactam. The remaining 37 isolates harboring *bla*_OXA_ demonstrated variable susceptibility to carbapenems but were uniformly susceptible to ceftazidime-avibactam. Among the 27 isolates unclassified by BD-CPO, 19 harbored *bla*_NDM_, *bla*_VIM_, or dual carbapenemase genes and were intermediate or resistant to all tested carbapenems and to ceftazidimeavibactam. The performance of BD-CPO compared with WGS is shown in Table 2. Carbapenem susceptibility patterns by carbapenemase gene family is shown in Table 3. Detailed isolate-level detection, classification, and antimicrobial susceptibility results are provided in Table S1 in the supplemental material.

**TABLE 2:** Performance of BD-CPO compared with WGS^*b*^^,*d*^

WGS Ambler class	No. of isolates	CPO^*a*^ positive, *n* (%)	Correct class assigned, *n* (%)	Unclassified, *n* (%)	Misclassified, *n* (%)	FN, *n* (%)
Class B only (NDM or VIM)	110	110 (100)	92 (83.6)	17 (15.5)	1 (0.9)	0
Class D only (OXA-48–like)	37	31 (83.8)	21 (56.8)	8 (21.6)	2 (5.4)	6 (16.2)
Dual carbapenemases (NDM + OXA or NMD + VIM)	14	14 (100)	0	2 (14.3)	12^*c*^ (85.7)	0
Total	161	155 (96.3)	113 (70.2)	27 (16.8)	15 (9.3)	6 (3.7)

^*a*^ CPO, carbapenemase-producing organism detection result generated by BD-CPO.

^*b*^ Only one out of two Ambler classes was assigned.

^*c*^ False negatives occurred exclusively among isolates harboring OXA-48–like enzymes.

^*d*^ FN, false negative; *n*, number; NDM, New Delhi metallo-β-lactamase; OXA-48–like, OXA-48 family carbapenemases; VIM, Verona integron-encoded metallo-β-lactamase; WGS, whole-genome sequencing.

**TABLE 3:** Carbapenem susceptibility patterns by carbapenemase gene family^*a*^

Carbapenemase category	No. of isolates	Ertapenem R/I, *n* (%)	Meropenem R/I, *n* (%)	Imipenem R/I, *n* (%)
Class B only (NDM or VIM)	110	110 (100)	110 (100)	110 (100)
Class D only (OXA-48–like)	37	24 (64.9)	6 (16.2)	17 (45.9)
Dual carbapenemases (NDM + OXA or NMD + VIM)	14	14 (100)	14 (100)	14 (100)

^*a*^ I, intermediate; *n*, number; NDM, New Delhi metallo-β-lactamase; OXA-48–like, OXA-48 family carbapenemases; R, resistant; VIM, Verona integron-encoded metallo-β-lactamase; WGS, whole﻿genome sequencing.

## DISCUSSION

CPE pose a global public health threat, underscoring the need for rapid and accurate laboratory detection of carbapenemase-producing organisms to guide effective therapy (17, 18). In this study, we evaluated the performance of NG-C5 and BD-CPO against WGS as the reference method for carbapenemase detection and classification in *Enterobacterales* isolates. The three methods evaluated in this study assess carbapenemase production at different biological levels. WGS identifies carbapenemase genes, NG-C5 detects carbapenemase enzymes at the protein level, and the BD-CPO assay evaluates functional enzyme activity. Therefore, discrepancies between methods may reflect differences in gene expression or enzymatic activity rather than assay performance alone and should be interpreted in a complementary rather than directly interchangeable manner.

NG-C5 demonstrated excellent accuracy, correctly identifying 99.4% of CPE isolates. Consistent with prior studies, it reliably detects common carbapenemase genes in both screening and clinical samples, including positive blood cultures, supporting its role as a first-line rapid diagnostic tool to enable timely patient management and implementation of infection control measures (19–22). The single observed discrepancy, in which an isolate harboring *bla*_NDM-5_ by WGS was misidentified as NDM + KPC by NG-C5, is consistent with a few previous studies that reported rare false-positive results using this immunochromatographic assay (23, 24). It is also worth highlighting the ability of the NG-C5 to detect multiple carbapenemase genes simultaneously (14 isolates in our study). The rapid detection of multiple carbapenemase genes directly in clinical specimens is clinically critical, as it can guide antimicrobial selection in patients with CPE infections, particularly in cases of bloodstream infection (25, 26).

The BD-CPO assay showed high sensitivity (96.3%) for detecting CPE isolates but was less accurate than NG-C5 (99.4%), correctly assigning the Ambler class in only 70.2% of isolates. Consistent with previous studies, the BD-CPO test demonstrates excellent sensitivity for detecting carbapenemase producers and can serve as a useful screening tool that is readily integrated into routine susceptibility workflows; however, its ability to accurately classify carbapenemases into the correct Ambler classes remains limited (27–29). The low specificity of the BD-CPO test and failure to classify CPEs into the correct Ambler class indicate that positive results require confirmation by a more specific method, such as NG-C5 (30, 31). CLSI guidelines also recommend the use of phenotypic methods such as mCIM/eCIM to differentiate MBLs from other carbapenemase classes (13). Early and accurate classification of CPE isolates enables clinicians to select targeted therapy, particularly with newer agents such as cefiderocol or β-lactam/β-lactamase inhibitor combinations such as ceftazidime-avibactam, meropenem-vaborbactam, and aztreonam-avibactam (32, 33). Although the BD-CPO assay demonstrated limitations in accurate Ambler class assignment, the associated phenotypic susceptibility profiles were largely consistent with expected resistance patterns. For example, isolates harboring MBLs (NDM and VIM) were uniformly resistant to carbapenems and ceftazidime-avibactam, which may still provide clinically actionable information and guide appropriate therapeutic strategies. In our cohort, 23% (37/161) of isolates were OXA-48-like producers, for which ceftazidime-avibactam represents a potential treatment option. In contrast, infections caused by the remaining 77% (124/161) of isolates producing Class B (MBLs) carbapenemases, including NDM- and VIM-type enzymes, may require alternative therapeutic strategies such as cefiderocol, aztreonam-avibactam, or combination therapy with ceftazidime-avibactam and aztreonam.

Six *E. coli* isolates producing OXA-48-type carbapenemases yielded false-negative results for carbapenemase detection by the BD-CPO assay. Notably, three of these isolates were susceptible to all tested carbapenems, while the remaining three were intermediate only to ertapenem. Similar studies have reported low but documented false-negative result rates associated with BD-CPO (31, 33, 34). OXA-48-like enzymes are known to exhibit relatively weak carbapenem hydrolytic activity, which can contribute to reduced detection sensitivity in functional assays such as BD-CPO (8, 9). This limitation likely explains the higher rate of false-negative and misclassification results observed for OXA-producing isolates in our study and highlights the challenges of directly comparing functional and immunochromatographic assays (31, 33, 34). These findings underscore the limitation of the BD-CPO test in isolates exhibiting low-level carbapenemase expression and reduced carbapenem MICs, such as OXA-48 producers. Therefore, the BD-CPO should be interpreted cautiously and in conjunction with carbapenem MIC values and susceptibility profiles for other β-lactam antibiotics, which may suggest carbapenemase activity.

The main strength of this study is the evaluation of a large number of clinical and screening CPE isolates with resistance genes characterized by WGS. However, several limitations should be acknowledged. First, the study was conducted at a single tertiary pediatric center with a specialized, high-risk patient population, which may limit the generalizability of our epidemiological findings to other healthcare settings or adult populations. Second, only WGS-confirmed CPE isolates were included, and the absence of a comparator group of CPE-negative isolates precluded calculation of diagnostic specificity for the evaluated assays. Third, the species distribution was uneven, with *E. coli* and *Klebsiella* spp. comprising the majority of isolates, potentially limiting extrapolation of performance data to other *Enterobacterales* species. Fourth, the discrepant NG-C5 result (false-positive KPC detection) was not re-evaluated by repeat testing as the isolate was no longer available, limiting our ability to determine whether this reflected a true assay limitation or a technical artifact. Finally, neither *bla*_KPC_ nor *bla*_IMP_ genes were detected in our cohort, limiting the assessment of NG-C5 and BD-CPO performance for these targets.

### Conclusion

In this study, we evaluated two rapid diagnostic assays for detection of CPE isolates using WGS as the reference standard. Both BD-CPO and NG-C5 demonstrated high sensitivity for CPE detection; however, NG-C5 showed superior performance, correctly identifying and classifying 99.4% of isolates, including simultaneous detection of dual carbapenemase genes in 14 isolates. Although the BD-CPO assay is integrated within the routine AST panel and provides phenotypic detection, its ability to accurately assign the correct Ambler class was limited, as a substantial proportion of isolates were misclassified or left unclassified. Rapid carbapenemase detection assays can significantly reduce turnaround time compared to conventional phenotypic approaches and facilitate timely antimicrobial optimization and infection control interventions. Nevertheless, these assays should be incorporated into comprehensive diagnostic stewardship strategies that include molecular confirmation and full antimicrobial susceptibility profiling to guide the management of patients with CPE infections and optimize surveillance programs to mitigate the spread of CPE within healthcare facilities.

## Data Availability

The data sets generated and/or analyzed during the current study are available from the corresponding author on reasonable request.

## SUPPLEMENTAL MATERIAL

Table S1 spectrum.00890-26-s0001.docx Detection and classification of CPE isolates by NG-C5 and BD-CPO compared with WGS, and antimicrobial susceptibility results.

ASM does not own the copyrights to Supplemental Material that may be linked to, or accessed through, an article. The authors have granted ASM a non-exclusive, world-wide license to publish the Supplemental Material files. Please contact the corresponding author directly for reuse.
