# Supplementary material for: Evaluation of BD Phoenix CPO Detect and NG-Test CARBA 5 for the detection and classification of carbapenemase-producing Enterobacterales using whole-genome sequencing as the reference standard
Source: Microbiol Spectr. 2026 Jun 18;14(8):e00890-26. doi: 10.1128/spectrum.00890-26 (PMC13436410; doi:10.1128/spectrum.00890-26)
Supplement: Supplementary file 1 [file spectrum.00890-26-s0001.docx]

**Table S1.** Detection and classification of CPE isolates by NG-C5 and BD-CPO compared with WGS, and antimicrobial susceptibility results.

| **Species** | **Number of isolates** | **WGS Results** | | **NG-C5**  **Results** | **BD-CPO Results** | | **Susceptibility Results** | | | |
| --- | --- | --- | --- | --- | --- | --- | --- | --- | --- | --- |
|  |  | **Type of genes** | **Ambler Class** |  | **CPO** | **Ambler Class** | **Erta** | **Mero** | **Imi** | **CZA** |
| ***Escherichia coli*** | 1 | NDM-5 | B | NDM | Pos | B | R | I | I | R |
|  | 65 | NDM-5 | B | NDM | Pos | B | R | R | R | R |
|  | 1 | NDM-1 | B | NDM | Pos | UC | R | R | R | R |
|  | 5 | NDM-5 | B | NDM* | Pos | UC | R | R | R | R |
|  | 2 | OXA-181 | D | OXA-48 | Pos | D | R | S | R | S |
|  | 2 | OXA-181 | D | OXA-48 | Pos | D | I | S | I | S |
|  | 2 | OXA-181 | D | OXA-48 | Pos | D | R | S | S | S |
|  | 1 | OXA-181 | D | OXA-48 | Pos | UC | R | S | I | S |
|  | 1 | OXA-181 | D | OXA-48 | Pos | UC | R | S | I | S |
|  | 3 | OXA-244 | D | OXA-48 | Pos | D | R | S | S | S |
|  | 1 | OXA-244 | D | OXA-48 | Pos | D | I | S | S | S |
|  | 1 | OXA-244 | D | OXA-48 | Pos | D | R | S | I | S |
|  | 3 | OXA-244 | D | OXA-48 | Pos | UC | R | S | S | S |
|  | 1 | OXA-244 | D | OXA-48 | Pos | UC | R | I | I | S |
|  | 1 | OXA-244 | D | OXA-48 | Pos | UC | I | S | S | S |
|  | 1 | OXA-244 | D | OXA-48 | Pos | B | R | S | I | S |
|  | 1 | OXA-244 | D | OXA-48 | Pos | B | R | S | S | S |
|  | 3 | OXA-244 | D | OXA-48 | Neg | ND | S | S | S | S |
|  | 1 | OXA-244 | D | OXA-48 | Neg | ND | I | S | S | S |
|  | 1 | OXA-484 | D | OXA-48 | Pos | D | R | S | S | S |
|  | 1 | OXA-484 | D | OXA-48 | Pos | D | R | S | I | S |
|  | 1 | OXA-484 | D | OXA-48 | Pos | D | I | S | R | S |
|  | 2 | OXA-484 | D | OXA-48 | Neg | ND | I | S | S | S |
|  | 1 | VIM-1 | B | VIM | Pos | B | I | R | R | R |
|  | 3 | NDM-5+OXA-181 | B+D | NDM+OXA-48 | Pos | D | R | R | R | R |
|  | 1 | NDM-5+OXA-181 | B+D | NDM+OXA-48 | Pos | B | R | R | R | R |
|  | 2 | NDM-5+OXA-244 | B+D | NDM+OXA-48 | Pos | D | R | R | R | R |
|  | 1 | NDM-5+OXA-244 | B+D | NDM+OXA-48 | Pos | D | R | I | I | R |
|  | 1 | NDM-5+OXA-244 | B+D | NDM+OXA-48 | Pos | UC | R | R | R | R |
| ***Klebsiella* spp*.*** | 12 | NDM-5 | B | NDM | Pos | B | R | R | R | R |
|  | 1 | NDM-5 | B | NDM | Pos | D | R | R | R | R |
|  | 9 | NDM-5 | B | NDM | Pos | UC | R | R | R | R |
|  | 5 | NDM-1 | B | NDM | Pos | B | R | R | R | R |
|  | 2 | NDM-1 | B | NDM | Pos | UC | R | R | R | R |
|  | 1 | OXA-48 | D | OXA-48 | Pos | D | R | R | R | S |
|  | 2 | OXA-48 | D | OXA-48 | Pos | D | R | I | I | S |
|  | 1 | OXA-48 | D | OXA-48 | Pos | D | R | I | R | S |
|  | 2 | OXA-48 | D | OXA-48 | Pos | D | R | S | R | S |
|  | 1 | OXA-232 | D | OXA-48 | Pos | D | R | S | S | S |
|  | 1 | OXA-232 | D | OXA-48 | Pos | UC | R | I | S | S |
|  | 1 | VIM-4 | B | VIM | Pos | B | R | I | R | R |
|  | 1 | NDM-5+OXA-232 | B+D | NDM+OXA-48 | Pos | D | R | R | R | R |
|  | 3 | NDM-5+OXA-48 | B+D | NDM+OXA-48 | Pos | D | R | R | R | R |
|  | 1 | NDM-5+OXA-181 | B+D | NDM+OXA-48 | Pos | D | R | R | R | R |
|  | 1 | NDM-5+VIM-1 | B+D | NDM+VIM | Pos | UC | R | R | R | R |
| ***Citrobacter* spp.** | 1 | NDM-5 | B | NDM | Pos | B | R | R | I | R |
|  | 2 | NDM-1 | B | NDM | Pos | B | R | R | R | R |
|  | 1 | NDM-7 | B | NDM | Pos | B | R | R | R | R |
| ***Enterobacter* spp*.*** | 3 | NDM-1 | B | NDM | Pos | B | R | R | R | R |

* One isolate harboring *bla*_NDM-5_ by WGS was misidentified as NDM+KPC by NG-C5

CZA, ceftazidime-avibactam; Erta, ertapenem; I, intermediate; Imi, imipenem; Mero, meropenem; ND, not determined; NDM, New Delhi metallo-β-lactamase; NG-C5, NG Test Carba 5; Neg, negative; OXA, **OXA-type β-lactamases;** Pos, positive; R, resistant; S, susceptible; UC, unclassified; VIM, Verona Integron-encoded Metallo-β-lactamase; WGS, whole-genome sequencing.
